# Diabetes Onset at 31–45 Years of Age is Associated with an Increased Risk of Diabetic Retinopathy in Type 2 Diabetes

**DOI:** 10.1038/srep38113

**Published:** 2016-11-29

**Authors:** Wenjun Zou, Lisha Ni, Qianyi Lu, Chen Zou, Minjie Zhao, Xun Xu, Haibing Chen, Zhi Zheng

**Affiliations:** 1 Department of Ophthalmology, Shanghai First People’s Hospital of Nanjing Medical University, Shanghai 200080, China; 2 Department of Ophthalmology, Nanjing Medical University Affiliated Wuxi Second Hospital, Wuxi 214002, China; 3 Department of Ophthalmology, Shanghai First People’s Hospital, Shanghai Jiao Tong University School of Medicine, Shanghai 200080, China; 4 Department of Ophthalmology, Lishui People’s Hospital, Lishui 323000, China; 5 Department of Ophthalmology, the First Affiliated Hospital of Soochow University, Suzhou 215006, China; 6 Department of Endocrinology and Metabolism, the Sixth People’s Hospital of Shanghai, Shanghai Jiao Tong University, Shanghai 200233, China

## Abstract

This hospital-based, cross-sectional study investigated the effect of age of diabetes onset on the development of diabetic retinopathy (DR) among Chinese type 2 diabetes mellitus (DM) patients. A total of 5,214 patients with type 2 DM who were referred to the Department of Ophthalmology at the Shanghai First People’s Hospital from 2009 to 2013 was eligible for inclusion. Diabetic retinopathy status was classified using the grading system of the Early Treatment Diabetic Retinopathy Study (ETDRS). Logistic and hierarchical regression analyses were used to identify independent variables affecting the development of DR. Upon multiple logistic regression analysis, patient age at the time of diabetes onset was significantly associated with development of DR. Further, when the risk of retinopathy was stratified by patient age at the onset of diabetes, the risk was highest in patients in whom diabetes developed at an age of 31–45 years (odds ratio [OR] 1.815 [1.139–2.892]; p = 0.012). Furthermore, when patients were divided into four groups based on the duration of diabetes, DR development was maximal at a diabetes onset age of 31–45 years within each group. A diabetes onset age of 31–45 years is an independent risk factor for DR development in Chinese type 2 DM patients.

The prevalence of type 2 diabetes mellitus (DM) has increased dramatically in young adults worldwide^1^. Currently, approximately one in three new cases of type 2 DM occur in patients younger than 18 years in the United States^2^. It is well known that diabetes is always associated with long-term complications, and diabetic retinopathy (DR) is one of the most frequent microvascular complications^3^. Several studies have described risk factors for DR; these include hypertension, the HbA1c level, age, the duration of diabetes, age at diabetes onset, and microalbuminuria (MA)^4^,^5^,^6^,^7^,^8^,^9^. Importantly, early onset type 2 DM patients with poor metabolic control have a higher prevalence of DR than patients with later onset^8^,^10^,^11^,^12^.

Wong *et al*.^8^ reported that patients with type 2 diabetes of long duration, diagnosed at <45 years of age, had a higher prevalence of DR than those diagnosed later, and another study^10^ found that patients diagnosed before the age of 40 years had higher mean concentrations of HbA1c and a higher prevalence of DR than those diagnosed at 40 years or older. These findings raise the questions of whether age group of diabetes onset at <45 years is associated with an increased DR risk, and whether the development of DR in a high-risk age group is independent of the duration of diabetes and glycemic control. To the best of our knowledge, no effort has been made to stratify the relationship between the age of onset of type 2 DM and DR prevalence.

We thus explored the role of age of DM onset on the development of DR among Chinese type 2 DM patients. The effect of age of diabetes onset on retinopathy status was examined to see if it would be independent of the duration of diabetes and the extent of glycemic control.

## Results

### Clinical characteristics of patients with and without DR

The demographic characteristics of all patients are shown in Table 1. Of the 5,214 patients, retinopathy was evident in 1,402 (26.9%). Patients with and without DR differed significantly in terms of age, duration of diabetes, age of onset, SBP, DBP, MA, eGFR, and HbA1c level. There was no between-group difference in any of WC, BMI, or FPG, TG, TC, HDL, LDL or C-reactive protein levels (Table 1).

### Risk factors affecting the development of DR

Upon univariate logistic regression analysis, age, age of onset, duration of diabetes, SBP, DBP, HbA1c, MA and eGFR were identified for the potential risk factors. Then we examined a correlation matrix to exclude correlated factors such as age and DBP. Therefore, age of onset, duration of diabetes, SBP, HbA1c, MA and eGFR should be permitted in the multiple logistic regression model. Upon mutiple logistic regression analysis, age of onset, duration of diabetes, SBP, HbA1c, and MA were independent risk factors for DR (Table 2, Model 1).

To stratify the risk of DR, patients were divided in terms of age at diabetes onset, as follows: ≤30 years, 31–45 years, 46–60 years, and ≥61 years. A diabetes onset age of 31–45 years was associated with an increased risk of DR. The 31–45-year age group was at the highest risk of DR, thus 1.815-fold (OR 1.815 [1.139–2.892]; p = 0.012) that of patients in the lowest age group (Table 2, Model 2).

### Effects of the age of onset of diabetes and diabetes duration on the prevalence of DR

Patients were divided into four groups according to duration of diabetes: ≤5 years, 6–10 years, 11–15 years, and >15 years. We calculated the prevalence of DR by age at diabetes onset (≤30 years, 31–45 years, 46–60 years, and ≥61 years) in the groups differing in terms of diabetes duration. When the duration was ≤5 years, 375 DR patients (16.3%) were evident among the total of 2,302 patients, the proportions of whom at each age of onset were 24/186 (12.9%), 93/483 (19.3%), 163/996 (16.4%), and 95/637 (14.9%). When the diabetes duration was 6–10 years, 408 DR patients (29.0%) were evident in the total of 1,405 patients, the proportions of whom at each age of onset were 10/49 (20.4%), 120/352 (34.1%), 179/665 (26.9%), and 99/339 (29.2%). When the diabetes duration was 11–15 years, 301 DR patients (39.3%) were present among the total of 766 patients, the proportions of whom at each age of onset were 7/28 (25.0%), 123/222 (55.4%),141/390 (36.2%), and 30/126 (23.8%). When the diabetes duration was >15 years, 318 DR patients (42.9%) were among the total of 741 patients, the proportions of whom at each age of onset were 17/38 (44.7%), 109/193 (56.5%), 168/416 (40.4%), and 24/94 (25.5%). Thus, patients with age of onset of 31–45 years had the highest prevalence of diabetic retinopathy, regardless of duration of diabetes, in this population. Furthermore, DR development was maximal at a diabetes onset age of 31–45 years after adjusting confounding factors such as SBP, HbA1c and MA (Table 3).

## Discussion

In this cross-sectional study of Chinese patients with type 2 diabetes, multiple logistic regression showed that the age at diabetes onset was significantly associated with the development of retinopathy, independent of the duration of diabetes, SBP, HbA1c level, and MA; these results are consistent with those of other studies^4^,^5^,^6^,^7^,^8^,^9^. We next divided all patients into four groups by age at diabetes onset, and found that an age at onset of 31–45 years was associated with the highest risk of DR, [1.815-fold (1.139–2.892-fold)] that of patients in the youngest age of onset group. Moreover, this was not affected by the duration of diabetes.

Previous studies suggested that early onset type 2 diabetes was more aggressive than late-onset disease^2^,^11^,^12^. In 2008, Wong *et al*.^8^ reported that an age at type 2 diabetes onset <45 years was associated with an increased inherent susceptibility to DR; the cited authors matched the duration of diabetes and the extent of glycemic control. Recently, a prospective cross-sectional study of an Asian cohort found that patients with younger-onset type 2 diabetes (diagnosed before the age of 40 years) had higher mean levels of HbA1c, and a greater prevalence of retinopathy, than those with late-onset diabetes (diagnosed at age ≥40 years)^10^. No study has yet addressed whether an age group at diabetes onset of <45 years was associated with a higher risk of retinopathy, independent of disease duration and the extent of hyperglycemia.

In the present study, we sought a definite relationship between age at diabetes onset and DR. We divided our patients into four groups by age at diabetes onset: ≤30, 31–45, 46–60, and ≥61 years. Next, based on the duration of diabetes, each group was divided into four subgroups: ≤5 years, 6–10 years, 11–15 years, and >15 years. We found that a diabetes onset age of 31–45 years was associated with an increased risk of DR development, independent of the duration of diabetes. However, the underlying mechanism remains unclear. Several possible explanations may be advanced. Some studies have found that the level of vascular endothelial growth factor (VEGF) in diabetes patients varies with age, and VEGF expression after stimulation is higher in younger than older patients^13^,^14^. When hyperglycemia is in play, VEGF promotes pathological retinal angiogenesis and fibrovascular proliferation during development of DR^15^,^16^. Therefore, we speculate that a gene such as that encoding VEGF may be more active in patients with diabetes onset at 31–45 years of age, predisposing such patients to development of DR. In addition, “metabolic memory” may contribute to an increased risk of DR. Patients developing diabetes at 31–45 years may prioritize personal and career development rather than their health, and are usually diagnosed after a long-term history of hyperglycemia. Many studies have shown that prolonged hyperglycemia causes injuries to the retinal vasculature that are not reversed even upon subsequent sustained glycemic control; such impairments may play pivotal roles in “metabolic memory”, rendering patients more susceptible to the complications of diabetes^17^,^18^,^19^,^20^. Last but not least, DM individuals diagnosed between 31–45 years of age are usually under high-level psychological pressure^21^,^22^,^23^. Such stress may explain why DR is more likely in patients in whom diabetes develops at an age of 31–45 years. Our findings have an important contribution to the monitoring and intervention in type 2 DM individuals in whom the diabetes onset age is 31–45 years of age with their working life at the peak of creation.

Our research had several limitations. Firstly, the work was performed in a single hospital and all patients were Chinese. Care should be taken when attempting to extrapolate this data to other patient populations. Secondly, a cross-sectional study cannot identify cause-and-effect relationships. Future multicenter, longitudinal longer-term studies are required to verify our results. Thirdly, Color photographs were acquired using a macula-centered view of fundus photgraph and supplementary fundus photgraphs of lesions. Standard 7-field fundus photographs should be used in our future study.

In summary, a diabetes onset age of 31–45 years is an independent risk factor for development of DR in type 2 DM patients. It is important to conduct stringent monitoring and intervention in type 2 DM individuals in whom the diabetes onset age is 31–45 years, to delay the development and progression of DR.

## Methods

### Study Design

A total of 5,214 inpatients with type 2 DM who were referred to the Department of Ophthalmology at the Shanghai First People’s Hospital and the Department of Endocrinology and Metabolism at the Sixth People’s Hospital of Shanghai from 2009 to 2013 was eligible for inclusion in this cross-sectional and retrospective study. DM was diagnosed using the criteria of the World Health Organization (WHO) [the WHO study group (1999)]. Exclusion criteria included type 1 DM which was excluded through measuring IA2Ab, GADAb, and IAA, gestational diabetes, acute complications of diabetes, thyroid problems, obstructive liver disease, advanced renal failure, heart disease, urinary tract infection, lung infection, cerebrovascular accident, or tuberculosis. The study was performed in accordance with the World Medical Association Declaration of Helsinki and was approved by the Institutional Review Board of the Shanghai First People’s Hospital. Written informed consent was obtained from all included patients.

### Data Collection

Medical record review was undertaken by a single researcher; demographic details, and physical and biochemical data, were recorded on a form. Demographic details included age, sex, age at diagnosis, duration of diabetes, and the general and ophthalmological medical histories. Physical examination included: systolic blood pressure (SBP), diastolic blood pressure (DBP), waist circumference (WC), and body-mass index (BMI). Laboratory data included the glycosylated hemoglobin (HbA1c) level; MA status; and the levels of fasting plasma glucose (FPG), triglycerides (TG), total cholesterol (TC), C-reactive protein, high-density lipoprotein (HDL), and low-density lipoprotein (LDL); and the estimated glomerular filtration rate (eGFR). The eGFR was calculated using the equation of the Modification of Diet in Renal Disease study^24^.

Each patient underwent a comprehensive ophthalmologic examination that included a review of ophthalmologic history, measurement of visual acuity and intraocular pressure (IOP), slit lamp biomicroscopy, and fundoscopic examination through dilated pupils via fundus photography and reading center to grade the retinopathy. Color photographs were acquired with a Zeiss Visucam 200 digital fundus camera (Carl Zeiss Meditec AG, Jena, Germany) using a macula-centered field of view. However, supplementary fundus photgraphs of lesions were taken for those who showed any evidence of DR. Diabetic retinopathy status was graded using the system of the Early Treatment Diabetic Retinopathy Study (ETDRS): 1) no DR; 2) nonproliferative disease (mild, moderate, severe); and, 3) proliferative. Whenever the two eyes were graded differently, the more advanced was chosen.

### Statistical Analysis

Patients were divided into two groups based on the presence or absence of DR. Descriptive statistics (means ± standard deviations [SDs]) of normally distributed variables, and geometric means with 95% confidence intervals (CIs) of non-normally distributed variables, were calculated to identify the characteristics of each group. Normally distributed data were compared between groups using an unpaired t-test, and non-normally distributed data using the Mann-Whitney U test. The relative contributions of covariates to DR risk were analyzed via logistic regression with stepwise exclusion of parameters. Multiple logistic regression analyses featured DR as the dependent parameter, and various risk factors identified upon univariate regression analysis as independent parameters. Hierarchical regression analysis was used to derive the relative contributions of risk factors for DR. Odds ratios (ORs) and 95% CIs are presented. All data were analyzed using Statistical Package for the Social Sciences (SPSS) software version 19.0 (SPSS, Los Angeles, CA). All reported p values are two-tailed, and a p value ≤ 0.05 was considered to indicate statistical significance.

## Additional Information

**How to cite this article**: Zou, W. *et al*. Diabetes Onset at 31–45 Years of Age is Associated with an Increased Risk of Diabetic Retinopathy in Type 2 Diabetes. *Sci. Rep.* **6**, 38113; doi: 10.1038/srep38113 (2016).

**Publisher's note:** Springer Nature remains neutral with regard to jurisdictional claims in published maps and institutional affiliations.

## Figures and Tables

**Table 1:** Clinical characteristics of type 2 diabetes mellitus patients with or without diabetic retinopathy (DR).

Variable	NO DR	DR	P value
Cases (M/F)	2,198/1,614	741/661	
Age (years)	58.2 ± 14.0	60.0 ± 12.0	<0.001
Age of onset (years)	52.0 ± 13.1	49.5 ± 11.3	<0.001
Duration of diabetes (years)	6.3 ± 5.8	10.5 ± 6.9	<0.001
SBP (mmHg)	130.0 ± 16.6	135.9 ± 19.4	<0.001
DBP (mmHg)	79.4 ± 9.6	80.6 ± 9.9	0.001
HbA1c (%)	9.0 ± 2.4	9.2 ± 2.1	0.008
MA (mg/24 h)	9.6 [3.3–209.8]	17.1 [4.0–1,602.8]	<0.001
FPG (mmol/L)	8.3 ± 3.0	8.6 ± 3.2	0.053
TG (mmol/L)	1.9 ± 1.8	1.8 ± 1.4	0.058
TC (mmol/L)	4.7 ± 1.1	4.8 ± 1.2	0.272
C-reactive protein level (mg/L)	1.1[0.2–13.9]	1.1 [0.2–20.2]	0.086
HDL (mmol/L)	1.1 ± 0.3	1.2 ± 0.3	0.059
LDL (mmol/L)	3.1 ± 1.0	3.1 ± 1.0	0.798
BMI (kg/m^2^)	24.8 ± 3.6	24.7 ± 3.6	0.692
Waist circumference (cm)	88.4 ± 10.6	88.3 ± 10.6	0.695
eGFR (ml·min^−1^·1.73 m^2^)	108.3 ± 36.5	104.9 ± 39.2	0.005

SBP: systolic blood pressure; DBP: diastolic blood pressure; WC: waist circumference; HbA1c: glycosylated hemoglobin; MA: microalbuminuria; FPG: fasting plasma glucose; TG: riglycerides; TC: total cholesterol; HDL: high-density lipoprotein; LDL: low-density lipoprotein; BMI: body-mass index; eGFR: estimated glomerular filtration rate. Data are expressed as means ± SDs or as geometric means [with 95% confidence intervals (CIs)].

**Table 2:** Risk factors by the presence or absence of DR as assessed via multiple logistic regression.

Variable	OR [95%CI]	P value	β
Model 1			
Age of onset (years)	0.985[0.977–0.992]	<0.001	−0.016
Duration of diabetes (years)	1.109 [1.093–1.125]	<0.001	0.103
SBP (mmHg)	1.014 [1.009–1.019]	<0.001	0.014
HbA1c (%)	1.064 [1.025–1.105]	0.001	0.062
MA (mg/24 h)	1.000 [1.000–1.000]	<0.001	0.000
eGFR (ml·min^−1^·1.73 m^2^)	0.998 [0.996–1.001]	0.186	−0.002
Model 2			
Age groups of onset		<0.001	
≤30 (years)	1 (reference)	—	—
31–45 (years)	1.815 [1.139–2.892]	0.012	0.596
46–60 (years)	0.574 [0.719–1.812]	0.574	0.132
≥61 (years)	0.724 [0.667–1.789]	0.724	0.089
Duration of diabetes (years)	1.110 [1.094–1.126]	<0.001	0.104
SBP (mmHg)	1.014 [1.009–1.019]	<0.001	0.014
HbA1c (%)	1.066 [1.026–1.107]	0.001	0.064
MA (mg/24 h)	1.000 [1.000–1.000]	<0.001	0.000
eGFR (ml·min^−1^·1.73 m^2^)	0.999 [0.996–1.001]	0.359	−0.001

DR: diabetic retinopathy; SBP: systolic blood pressure; HbA1c: glycosylated hemoglobin; MA: microalbuminuria; eGFR: estimated glomerular filtration rate.

**Table 3:** Effects of the age of onset of diabetes and diabetes duration on the prevalence of DR.

Age groups of onset	Duration of diabetes							
	≤5 (years)		6–10 (years)		11–15 (years)		>15 (years)	
Model 1	The prevalence of DR (DR/Total,%)							
≤30 (years)	24/186	12.9	10/49	20.4	7/28	25.0	17/38	44.7
31–45 (years)	93/483	19.3	120/352	34.1	123/222	55.4	109/193	56.5
46–60 (years)	163/996	16.4	179/665	26.9	141/390	36.2	168/416	40.4
≥61 (years)	95/637	14.9	99/339	29.2	30/126	23.8	24/94	25.5
Model 2	The prevalence of DR(%, adjust SBP, HbA1c, MA)							
≤30 (years)	15.0		12.0		22.0		50.0	
31–45 (years)	21.0		32.0		51.0		61.0	
46–60 (years)	15.0		29.0		34.0		43.0	
≥61 (years)	16.0		31.0		24.0		32.0	

The prevalence of retinopathy was calculated per group in terms of diabetes onset age, with reference to diabetes duration, for all 5,214 patients. DR: diabetic retinopathy; SBP: systolic blood pressure; HbA1c: glycosylated hemoglobin; MA: microalbuminuria.
