# Corrigendum: Diabetes Onset at 31–45 Years of Age is Associated with an Increased Risk of Diabetic Retinopathy in Type 2 Diabetes

**DOI:** 10.1038/srep46839

**Published:** 2017-05-26

**Authors:** Wenjun Zou, Lisha Ni, Qianyi Lu, Chen Zou, Minjie Zhao, Xun Xu, Haibing Chen, Zhi Zheng

Scientific Reports 6: Article number: 3811310.1038/srep38113; published online: 11 29 2016; updated: 05 26 2017

The original version of this Article incorrectly included Haibing Chen as a corresponding author. This error has now been corrected in the PDF and HTML versions of this Article.

